# Effect of xylo-oligosaccharides on intestinal bacterial diversity in mice with spleen deficiency constipation

**DOI:** 10.3389/fmicb.2024.1474374

**Published:** 2024-10-15

**Authors:** Xiang Ao, Zeying Zhang

**Affiliations:** ^1^Department of Pharmacy, Jiangxi College of Traditional Chinese Medicine, Fuzhou, China; ^2^Food and Health Research Institute, Wuchang Institute of Technology, Wuhan, China

**Keywords:** xylo-oligosaccharides, intestinal bacterial diversity, mice, spleen deficiency constipation, 16s rDNA sequencing

## Abstract

**Objective:**

To explore the effect of xylo-oligosaccharides on intestinal bacterial diversity in mice with spleen deficiency constipation.

**Methods:**

The 16S rDNA sequencing was used to identify microbiota composition in four groups, including the normal group (NG), the model group with spleen-deficiency constipation (SDC), XOS treated groups that include XOS1 groups treated XOS 0.05 g/mL•d, and XOS2 group treated XOS 0.1 g/mL•d. Chao1 and Shannon were used to conduct gut microbes diversity analysis. Linear discriminant analysis coupled with effect size measurements (LEfSe) was used to identify signature gut microbiota, and phylogenetic investigation of communities by reconstruction of unobserved states (PICRUSt) was used to predict the function of altered gut microbiota.

**Results:**

Veen map indicated 245 common OTUs were identified from four groups. Especially, 9, 3, 0, and 19 unique OTUs were identified in NG, SDC, XOS1, and XOS2 groups, respectively. The Shannon index was evidently higher in NG group than in the other three groups (*p* < 0.05). We identified the occurrence of dominant bacterial groups including Bacteroidetes (25.5 ~ 49.9%), Firmicutes (25.4 ~ 39.3%), Proteobacteria (12.5 ~ 24.9%), Deferribacteres (1.6 ~ 19.2%), Cyanobacteria (0.3 ~ 1.8%), Verrucomicrobia (0.02 ~ 1.6%), Actinobacteria (0.01 ~ 0.5%), and Tenericutes (0.03 ~ 0.09%) at the four groups. The XOS2 group was characterized by a higher abundance of *Peptostreptococcaceae*, *Intestinibacter*, *Aerococcaceae*, and *Facklamia*. XOS1 group enriched in *Deferribacteres*, *Mucispirillum*, *Deferribacterales*, *Deferribacteres*, *Lachnoclostridium*, *Rhodospirillaceae*, and *Rhodospirillales*. Meanwhile, the SDC mice showed dramatic enrichment in *Rikenellaceae*, *Lachnospiraceae*, *Rikenellaceae*, *Roseburia*, and *Alistipes*, which were highly abundant in the NG group. XOS fed-mice evidently increase *Deferribcteres* abundance compared with NG and SDC groups. However, the abundance of *Rikenellaceae* was significantly reduced in XOS1 and XOS2 groups compared with NG and SDC groups. We identified that altered gut microbiotas by XOS treatment were associated with various metabolic pathways, including organismal systems, metabolism, human diseases, genetic information processing, and cellular processes.

**Conclusion:**

Our research indicated that XOS has the potential to recover intestinal bacteria and contribute to the treatment of spleen deficiency constipation.

## Introduction

Spleen deficiency, known as “Spleen Qi Deficiency” in Traditional Chinese Medicine (TCM), is a concept that refers to a state where the spleen’s functional energy, known as “Qi,” is weakened. In TCM, the spleen is considered a critical organ for digestion, energy production, and overall vitality. It is associated with the transformation and transportation of food, absorption of nutrients, and the production of Qi and blood. When spleen function is weak, it can lead to a slowdown in the digestive process, resulting in constipation (Du Toit, 2019). A research found that the formula could modulate the composition of intestinal flora and alleviate spleen deficiency in rats, suggesting that the regulation of gut microbiota by traditional Chinese medicine could be a potential therapeutic approach for spleen deficiency-related constipation (De Maesschalck et al., 2015). In modern society, constipation is a common physiological state that causes gastrointestinal dysfunction (Ishaq et al., 2021; Zhu et al., 2018), affecting about 27% of global population (Cheng et al., 2003). Recent studies have proved that the imbalance of intestinal ecology in a patient with constipation was related to the variety and number of intestinal microbiota decrease, and the number of harmful pathogenic bacteria significantly increased, including aerobic bacteria, fungi and *Escherichia coli*, and a significant reduction in anaerobic bacteria, bacteroides and bifidobacteria (Van Laere et al., 2000; Zafar and Saier, 2021). In children with functional constipation, the dominant intestinal bacteria are mainly opportunistic pathogens such as bacteroides, escherichia, clostridium and fungi (Sanders et al., 2019). Studies have shown that the changes in the microbes and the enzyme activities in the intestine of mice with spleen-deficiency constipation were significant, and microbes such as colibacillus, bifidobacteria and lactobacilli were all proliferated (Zofou et al., 2019). Furthermore, many traditional Chinese medicine prescriptions had a good effect on the treatment of spleen-deficiency constipation, which can regulate the balance of intestinal microflora and intestinal enzyme activity through different pathways (Wu et al., 2022; Morgan et al., 2022).

Plant-based prebiotics are gaining significant attention for their potential health benefits and role in disease treatment. Water extract from silver fir (*Abies alba*) wood is effective in different pathological conditions, such as diabetes, cardiovascular diseases and psoriasis (Corominas-Murtra et al., 2014). Cocoa polyphenols can modulate the composition of the gut microbiota by exerting prebiotic mechanisms and enhancing the growth of beneficial gut bacteria (Pitta et al., 2014). As the hydrolysis product of xylan, xylooligosaccharides (XOS) are oligomers of *β*-1, 4-linked xylose residues with various substituents, including acetyl, phenolic, and uronic acid. They are found in fruits, vegetables, bamboo, honey, milk, and xylan-rich lignocellulosic material obtained from agricultural, forestal, and industrial waste (Chey et al., 2023; Yang et al., 2015). It is a type of prebiotic that has gained attention for its potential health benefits and role in disease treatment and general well-being. In addition, different bacteria inhabiting human gut, such as *Bifidobacterium* spp., *Bacteroides* spp., and *Clostridium* spp., utilize different polysaccharides and oligosaccharides as a source of energy (Lewis et al., 2019). Bifidobacteria, a genus of probiotic bacteria, play crucial roles in the human gut microbiota, contributing to various aspects of health and disease treatment. Studies have shown that XOS supplementation can alter the gut microbiota composition, increasing the abundance of bifidobacteria and potentially reducing the levels of harmful bacteria (Wen et al., 2022). Bacteroides species are essential for a healthy gut microbiota, contributing to nutrient metabolism, immune modulation, and protection against pathogens (Thomas et al., 2011). The interaction between bacteroides species and xylooligosaccharides (XOS) in the gut is a significant aspect of maintaining a balanced gut microbiome and promoting host health, including modulation of the immune response, and production of short-chain fatty acids (Edgar, 2010; Ding et al., 2021). Moreover, XOS is non-carcinogenic, stimulates bacterial growth and fermentation, and improves intestinal mineral absorption. They also possess antioxidant, antiallergenic, antimicrobial, immunomodulatory and selective cytotoxic activity, as well as blood and skin health-related effects (Yang et al., 2015; Tu et al., 2020). Furthermore, a report found the potential prebiotic effect of XOS from sugarcane, by its capacity to generate butyrate and increase the health-beneficial bifidobacteria (Costa et al., 2012).

In the present study, the effect of xylo-oligosaccharides on intestinal bacterial diversity in mice with spleen deficiency constipation was explored, and microbiota composition was analyzed using sequencing of the V3-V4 region of the 16S rRNA gene. We investigated the effect of XOS on the treatment of spleen-deficiency constipation in mice to elucidate the therapeutic mechanism of XOS, with a particular focus on the relationship between changes in intestinal microflora and constipation. Our findings suggest that XOS supplementation May be helpful for preventing spleen-deficiency constipation via modulating the composition of the gut microbiota.

## Materials and methods

### Reagents

The mixture of xylo-oligosaccharides (90%) was purchased from VETEC Company. Folium sennae was purchased from Hubei Shengdetang Decoction Co. LTD. Take sennae 500 g, plus 10 times the amount of boiling water for 10 min filtration, the filtrate in 75°C water bath evaporation reduced to 100% (1 g/mL crude drugs) water decoction. 4°C refrigerator spare, thawing to 25–30°C before use.

### Animals and procedures

Mice were purchased from Hubei Experimental Animal Research Center with license number (SCXK (e) 2015-0018). All procedures involving animals were performed according to protocols approved by the Institutional Animal Care and Use Committee of Hunan University of Chinese Medicine. The experiment included an equal number of male and female mice. All the mice were fed for 2 days to adapt to the condition. The mice were randomly divided into 4 groups: the normal group (NG), the model group with spleen-deficiency constipation (SDC), XOS treated groups that include XOS1 groups treated XOS 0.05 g/mL•d, and XOS2 group treated XOS 0.1 g/mL•d. The normal group’s mice were administered with sterile water (0.8 mL/mouse) twice a day. In addition, a Senna leaf water decoction, a traditional herbal preparation made from the leaves of the Senna plant, which is known for its laxative properties, is used to create an animal model that simulates spleen deficiency (Chung et al., 2020). The mice of the other groups were treated with senna water decoction (0.8 mL/mouse) by mouth, twice a day, for 7 days, then the diet of the mice was controlled by irregular eating for 8 days to prepare the model with spleen deficiency constipation. The intestine contents were collected after mice were euthanized by cervical dislocations, and then were frozen immediately and stored at −20°C.

### DNA extraction

DNA was extracted following standard procedures. 2.0 g of intestinal faces was weighed in a sterile environment and was homogenized in 5 mL of 0.1 mol/L phosphate buffer solution (PBS), followed by centrifugation at 200 g for 2 min. After washing twice with PBS, the whole supernatant was transferred into new tubes and centrifuged for 8 min at 10,000 × g. The sediment was gathered, washed once with PBS, twice with acetone, and three times with PBS, and then resuspended in 4 mL TE buffer. Five hundred μL of spreadhead were added with 45 μL TE buffer, 5 μL proteinase K, and 20 μL lysozyme, and homogenized in 1.5 mL germ-free EP tubes. Samples were incubated at 37°C for 30 min, and then mixed with 30 μL of 10% SDS, followed by incubation at 37°C for 40 min with turning upside down once every 10 min. Afterward, 100 μL of 5 mol/L NaCl and 80 μL of CTAB/NaCl were added and mixed well. The mixture was reacted at 65°C for 10 min. An equal volume of Tris-saturated phenol–chloroform-isoamyl alcohol (25:24:1) was then added to the sample, mixed well, and centrifuged at 10,000 × g for 3 min. The supernatant was transferred to fresh sterile tubes, mixed with an equal volume of chloroform-isoamyl alcohol (24:1), and centrifuged at 10,000 × g for 3 min. The supernatant was transferred into fresh sterile tubes and mixed with an equal volume of chloroform-isoamyl alcohol (24:1) again. After centrifugation at 10,000 × g for 3 min, the supernatant was transferred into new sterile tubes, added with 1/10 volume of 3 mol/L sodium acetate and double volume of absolute ethyl alcohol, and was precipitated at-20°C overnight. Samples were centrifuged at 10,000 × g for 3 min. The acquired sediment was washed with 70% ethanol, dried, and eventually dissolved in 50 μL TE buffer.

### PCR amplification of 16S rDNA V3-V4 region and sequencing

The primers 341f (CCTACGGGNGGCWGCAG) and 805R (GACTACHVGGGTATCTAATCC) were used for PCR amplification of the V3-V4 region of the 16S rRNA gene. PCR mixture (25 μL) contained 2 μL 2.5 mmol/L dNTP Mixture, 5.0 μL 5 × Q5 Reaction Buffer, 5.0 μL 5 × Q5 high Enhancer, 1.0 μL 10 μmol/L 341f, 1.0 μL 10 μmol/L 805R, 2.0 μL 0.2 ng/μL template DNA, 0.25 μL 5 U/μL Q5 Polymerase, and 8.75 μL sterilized ddH2O. Cycling parameters were as follows: initial denaturation at 98°C for 30 s; denaturation at 98°C for 30 s, annealing at 50°C for 30 s and extension at 72°C for 30 s, repeated for 25 cycles; last cycle of final extension at 72°C for 5 min. Then, PCR products were detected by 2% Agarose gel electrophoresis. Ultimately, the amplified V3-V4 region of 16S rDNA was sequenced by Biomarker Technologies.

### Data analyses

Three methods were applied to analyze the variety of microorganisms by determining operational taxonomic unit (OTU), including community composition analysis (CCA) and alpha diversity analysis (ADA). OTU was developed to measure the last node in phylogenetic or population genetics (You et al., 2020). Literally, the genetic DNA was broken into the number of units and OTU according to the homogeneity. The analysis was conducted by clustering using the software Qiime (Zeyue et al., 2022; Sorrenti et al., 2020). While CCA tests genetic similarity at various levels, such as phylum, class, order, and so on, ADA is a method of measuring population density and variety by its Chao index and Shannon index, respectively. Higher Chao index indicates higher population density, and a higher Shannon index means higher diversity (Krajmalnik-Brown et al., 2015; Venema et al., 2020; Altschul et al., 1990). Different components are orthogonal to each other. It was conducted using package of R. The significant differences among three groups were determined using one-way ANOVA. Differences among groups were considered significant when *p* values < 0.05.

## Results

### Xylo-oligosaccharides treatment was shown to alter the richness and diversity of the intestinal microorganisms in mice

**Figure 1:**
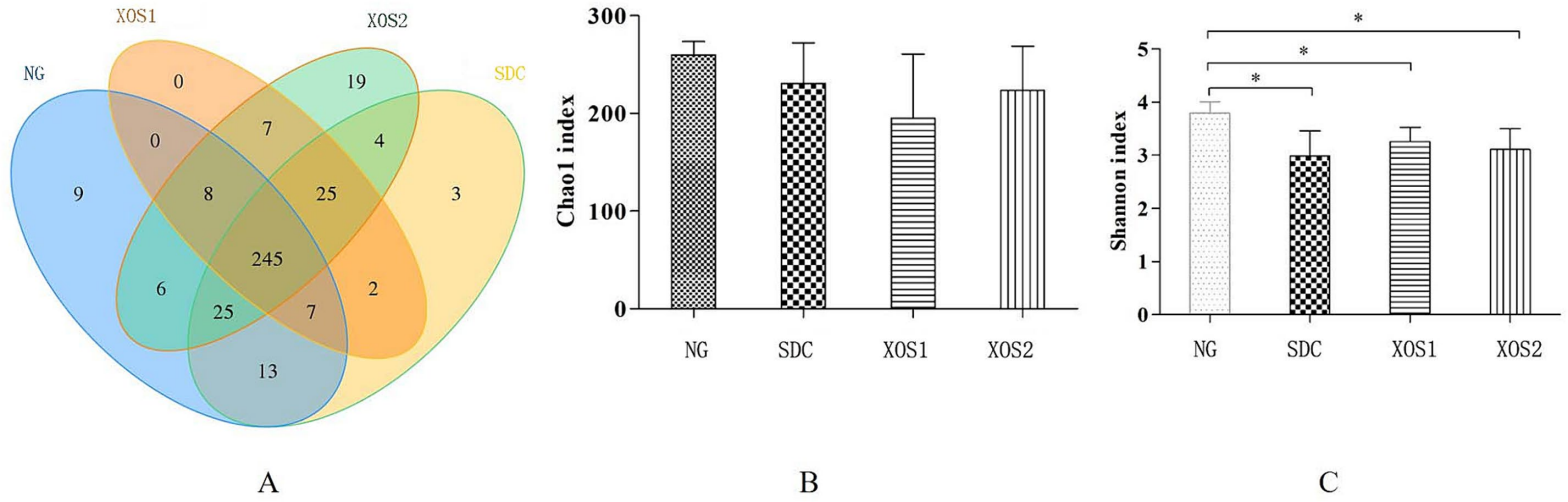
Comparison of the OTUs and Alpha diversity analysis in four groups. **(A)** Venn diagram of OTUs based on the sequences with over 97% similarity under a similar level of clustering. **(B,C)** Alpha diversity analysis of Chao and Shannon indexes, respectively. Compared with the NG, *p < 0.05.

In our study, for comparisons of the microbial diversity in different groups, we performed a diversity analysis, in which we identified 53,036–66,252 effective tags from 22 samples. The average length was 412–424 bp and GC content was 50.81–53.78%. The Q30 percentage was over 94.5%, which means good sequencing quality (Table 1). Furthermore, veen map indicated 245 common OTUs were identified from four groups. Especially, 9, 3, 0, 19 unique OTUs were identified in NG, SDC, XOS1, and XOS2 groups, respectively (Figure 1A). Meanwhile, Shannon and Chao 1 (Figures 1B,C) were used to estimate the richness and diversity of the intestinal microorganisms in mice. The shannon index was evidently higher in NG group than in the other three groups (*p* < 0.05). Compared with the other three groups, the normal group had the highest Chao1 index, however, it did not show any significance.

**Table 1:** The sample sequencing data processing results statistics.

Sample	PE reads	Raw tags	Clean tags	Effective tags	Avg-length (bp)	GC (%)	Q20 (%)	Q30 (%)	Effective (%)
NG01	80,043	75,204	67,916	59,129	420	51.7	97.27	94.73	73.87
NG02	80,084	74,988	67,848	66,252	418	52.86	97.37	94.85	82.73
NG03	80,016	75,854	69,403	67,629	416	52.81	97.36	94.86	84.52
NG04	79,899	72,399	63,905	62,202	421	53.46	97.21	94.55	77.85
NG05	79,861	74,976	68,139	65,758	417	53.78	97.42	94.89	82.34
NG06	79,983	74,190	66,610	61,393	418	53.0	97.32	94.79	76.76
SDC01	80,015	75,122	64,395	61,663	412	53.53	97.41	94.98	77.06
SDC02	79,862	74,079	66,153	62,367	424	53.25	97.22	94.58	78.09
SDC03	80,186	76,391	69,641	64,935	416	52.94	97.35	94.9	80.98
SDC04	79,985	76,359	69,918	63,427	414	50.95	97.36	94.92	79.3
XOS101	79,413	75,305	68,391	58,660	419	51.64	97.24	94.68	73.87
XOS102	80,117	75,753	68,945	60,201	419	52.28	97.27	94.76	75.14
XOS103	80,099	76,168	69,645	65,365	420	52.45	97.35	94.86	81.61
XOS104	79,993	76,232	69,943	64,556	416	51.71	97.36	94.9	80.7
XOS105	80,009	75,785	64,245	53,036	421	51.23	97.27	94.79	66.29
XOS201	80,063	75,716	68,781	61,236	420	52.05	97.27	94.71	76.48
XOS202	79,849	75,082	67,911	62,301	418	52.19	97.3	94.78	78.02
XOS203	79,890	76,250	69,484	58,281	419	50.61	97.25	94.76	72.95
XOS204	79,898	76,389	70,566	68,735	413	52.07	97.45	95.12	86.03
XOS205	79,445	75,859	69,039	62,806	420	52.58	97.32	94.95	79.06
XOS206	79,897	75,105	68,139	63,453	418	52.45	97.35	94.84	79.42
XOS207	79,741	75,965	69,134	58,002	421	50.81	97.2	94.66	72.74

### Impact of XOS treatment on gut microbial composition in mice

**Figure 2:**
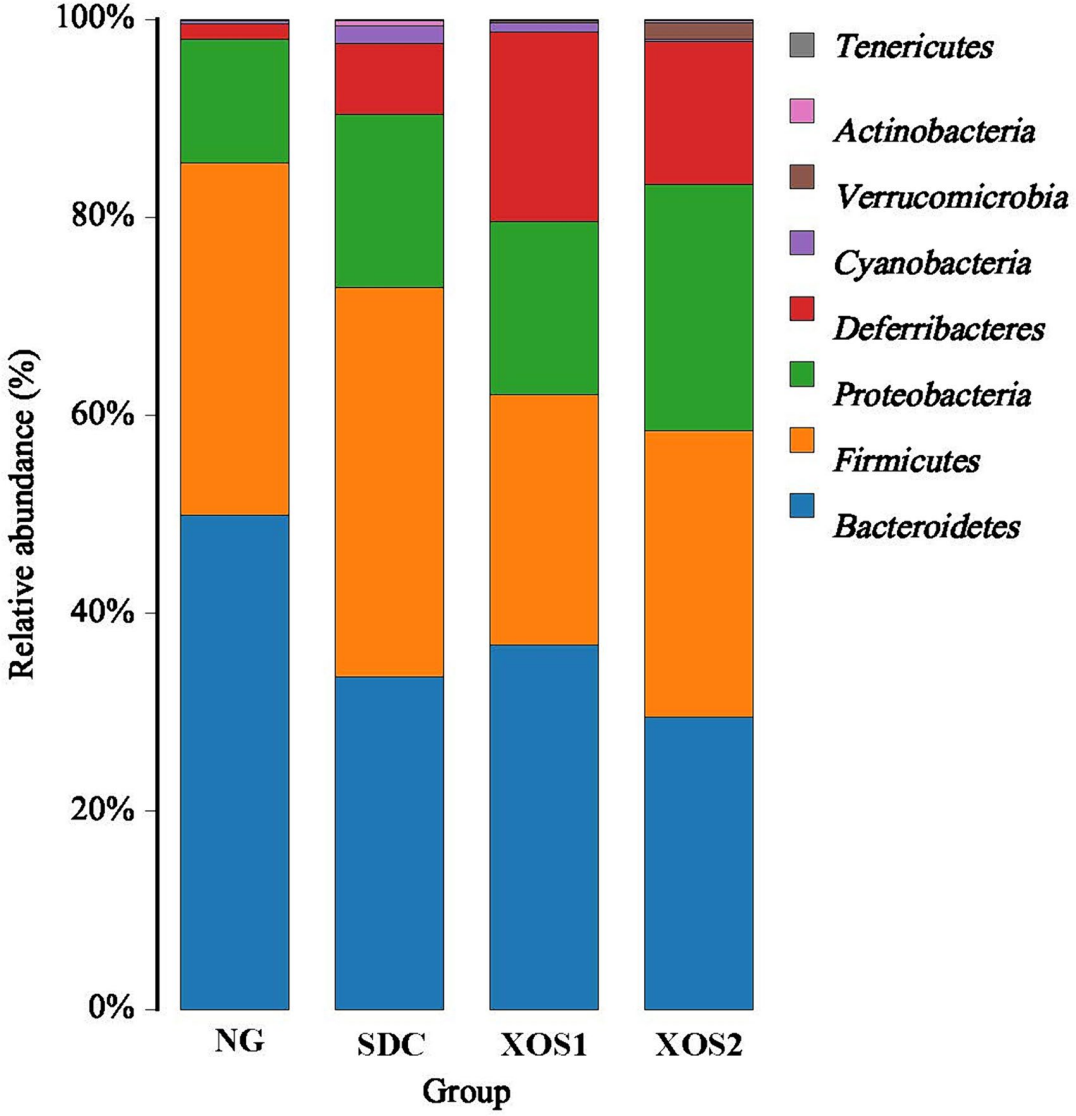
Community composition of fecal microbiota in phylum levels.

**Figure 3:**
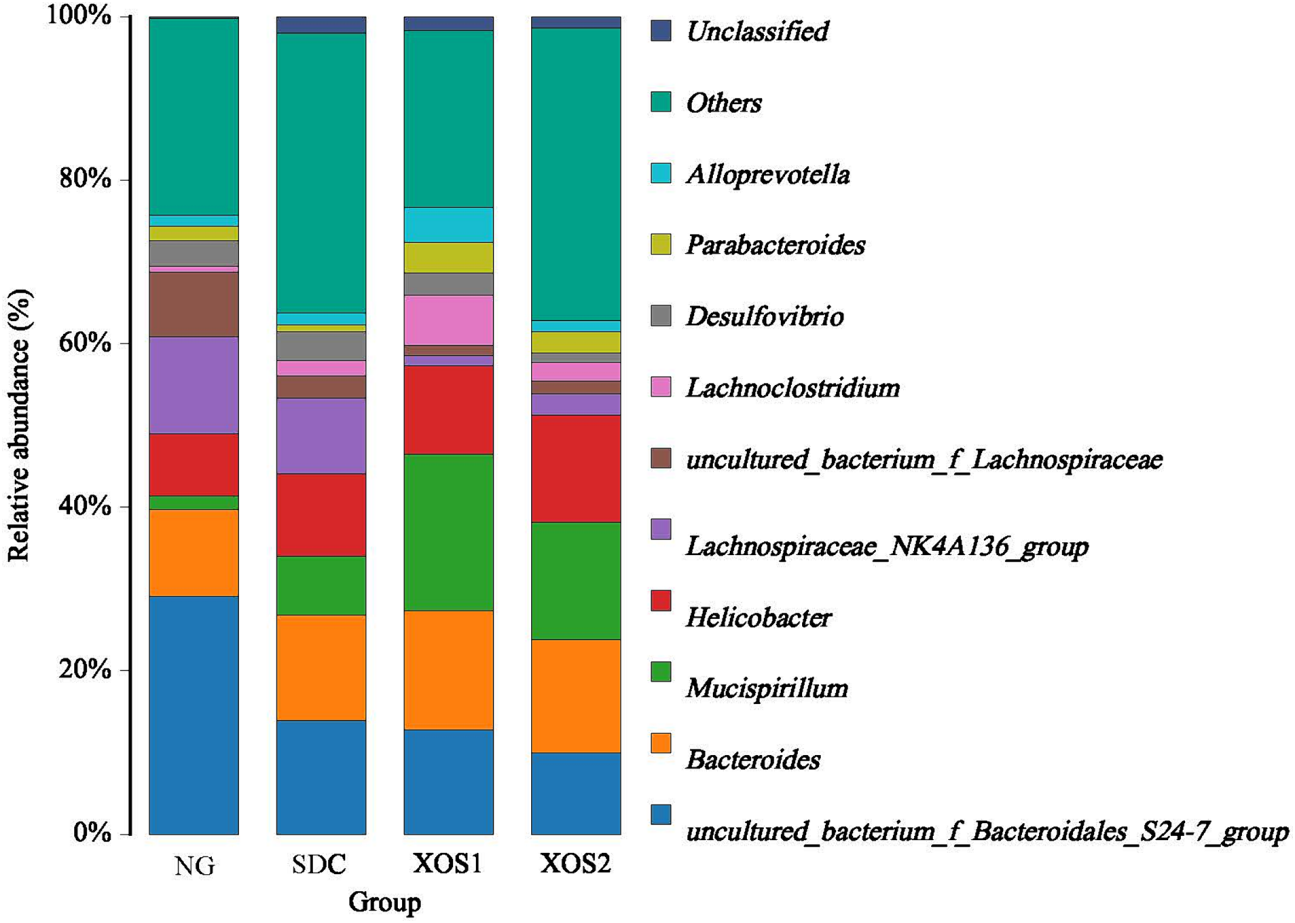
Community composition of fecal microbiota in genus levels.

In the phyla level, we identified the occurrence of dominant bacterial groups including Bacteroidetes (25.5 ~ 49.9%), Firmicutes (25.4 ~ 39.3%), Proteobacteria (12.5 ~ 24.9%), Deferribacteres (1.6 ~ 19.2%), Cyanobacteria (0.3 ~ 1.8%), Verrucomicrobia (0.02 ~ 1.6%), Actinobacteria (0.01 ~ 0.5%), and Tenericutes (0.03 ~ 0.09%) at the four groups. In NG, XOS1, and XOS2 groups, the bacteria from Bacteroidetes (49.9, 36.8, and 29.5%) were the most abundant, followed by Firmicutes (35.6, 25.4, and 28.9%) and Proteobacteria (12.5, 17.4, and 24.9%). In SDC group, the bacteria from Firmicutes (39.3%) were the most abundant, followed by Bacteroidetes (33.6%) and Proteobacteria (17.5%). In the NG group, only the Bacteroidetes were significantly higher than other groups (*p* < 0.05). The relative percentage of Deferribacteres was similar among NG, XOS1, and XOS2 groups, which was significantly higher than that of SDC (*p* < 0.05). However, SDC group had the highest relative abundance of Cyanobacteria and Verrucomicrobia (*p* < 0.05) (Figure 2; Table 2). At the genus level, in the NG group, *uncultured_bacterium_f_Bacteroidales_S24-7_*group, *Helicobacter* and *Bacteroides* were the dominant genus (29.1, 11.9, and 10.6%); in the SDC group, *uncultured_bacterium_f_Bacteroidales_S24-7_group*, *Bacteroides* and *Helicobacter* were the dominant genus (13.9, 12.9, and 10.1%); in XOS1 and XOS2 groups, the *Mucispirillum* was the most abundant genus (19.2 and 14.4%) (Figure 3; Table 3).

**Table 2:** Relative abundance of the gut bacteria at the phylum level.

Phylum	NG	SDC	XOS1	XOS2
Bacteroidetes	0.499065	0.335595	0.367603	0.295289
Firmicutes	0.35586	0.393286	0.253663	0.289478
Proteobacteria	0.124689	0.174989	0.174134	0.248663
Deferribacteres	0.016322	0.071888	0.19201	0.144211
Cyanobacteria	0.003208	0.017906	0.009196	0.002836
Verrucomicrobia	0.000399	0.000177	0.001892	0.015912
Actinobacteria	0.000113	0.005207	0.00118	0.002728
Tenericutes	0.000345	0.000952	0.000321	0.000883

**Table 3:** Relative abundance of the gut bacteria at the genus level.

	NG	SDC	XOS1	XOS2
Uncultured_bacterium_f_Bacteroidales_S24-7_group	0.290696643	0.139084113	0.127041923	0.098910896
Bacteroides	0.106427923	0.128960771	0.145851055	0.138808478
Mucispirillum	0.016322338	0.071888217	0.192010242	0.144210811
Helicobacter	0.076329027	0.100598765	0.108271851	0.130672937
Lachnospiraceae_NK4A136_group	0.118716599	0.092670721	0.011817551	0.025933428
Uncultured_bacterium_f_Lachnospiraceae	0.078526724	0.027368399	0.012976304	0.015518822
Lachnoclostridium	0.008255015	0.018165833	0.061648294	0.022721004
Desulfovibrio	0.030825774	0.035879082	0.026564534	0.012183807
Parabacteroides	0.017103817	0.008042491	0.03816943	0.025833127
Alloprevotella	0.014035914	0.014904099	0.042635188	0.012958356
Others	0.240797996	0.342903516	0.216061974	0.358442991
Unclassified	0.00196223	0.019533993	0.016951654	0.013805343

### XOS treatment induces a significant difference between *Deferribcteres* and *Rikenellaceae*

**Figure 4:**
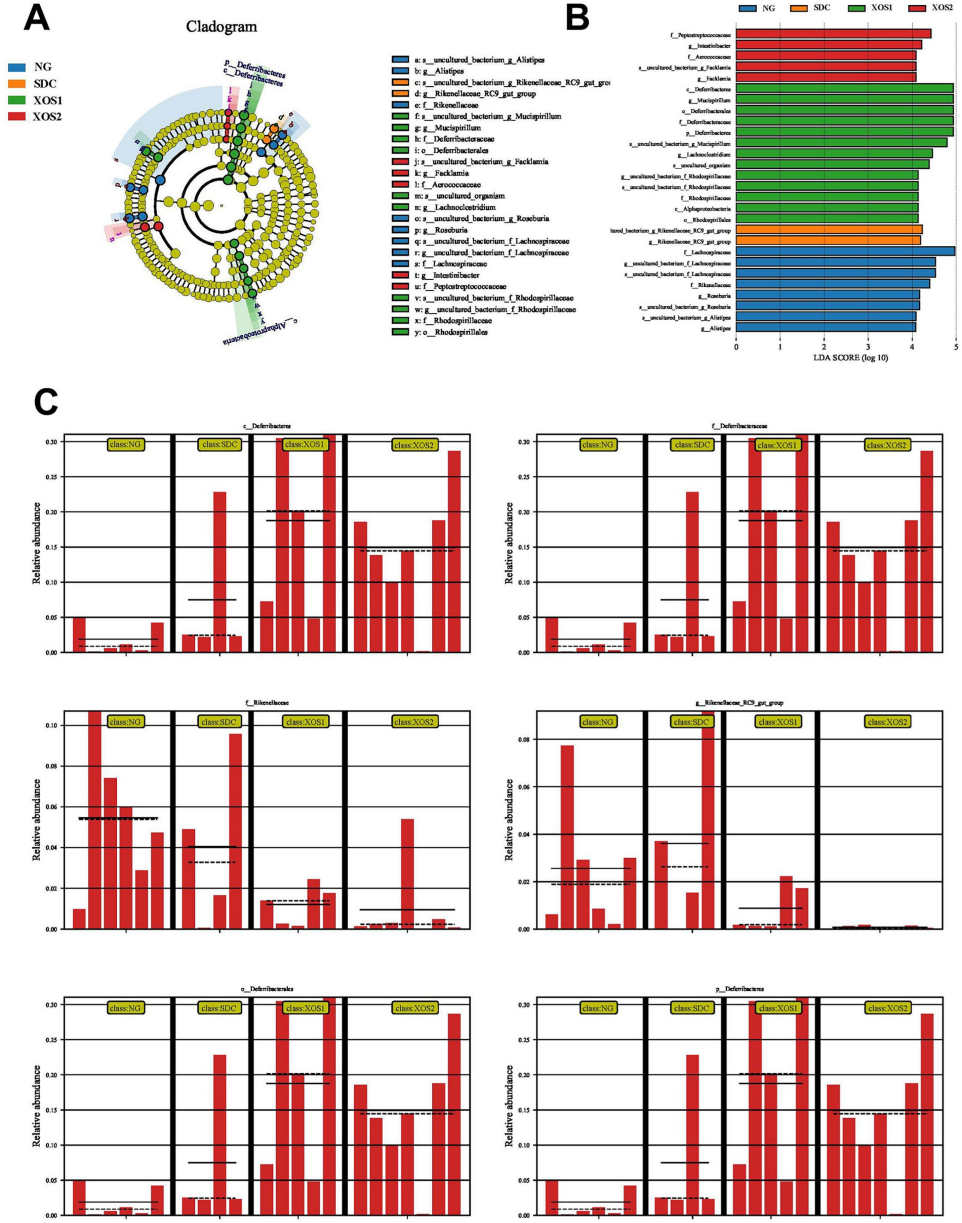
XOS-induced alterations in gut microbiota. Graphical phylogenetic analysis of gut microbiota alterations among 3 groups **(A)**. LDA scores of the differentially abundant bacteria and abundance heatmap of the bacteria in the four groups **(B)**. XOS-induced differentially altered gut microbiota **(C)**.

The LEfSe analysis indicated shifts in specific microbial families in the four groups (Figure 4). Evidently, a higher abundance of *Peptostreptococcaceae*, *Intestinibacter*, *Aerococcaceae*, and *Fack lamia* characterized the XOS2 group. XOS1 group enriched in *Deferribacteres*, *Mucispirillum, Deferribacterales, Deferribacteres, Lachnoclostridium, Rhodospirillaceae*, and *Rhodospirillales*. Meanwhile, the SDC mice showed dramatic enrichment in *Rikenellaceae*. *Lachnospiraceae, Rikenellaceae, Roseburia*, and *Alistipes* were highly abundant in the NG group. Furthermore, XOS fed-mice evidently increase *Deferribcteres* abundance compared with NG and SDC groups. However, the abundance of *Rikenellaceae* was significantly reduced in XOS1 and XOS2 groups compared with NG and SDC groups.

### Functional prediction of altered gut microbiota in xylo-oligosaccharides treated progression

**Figure 5:**
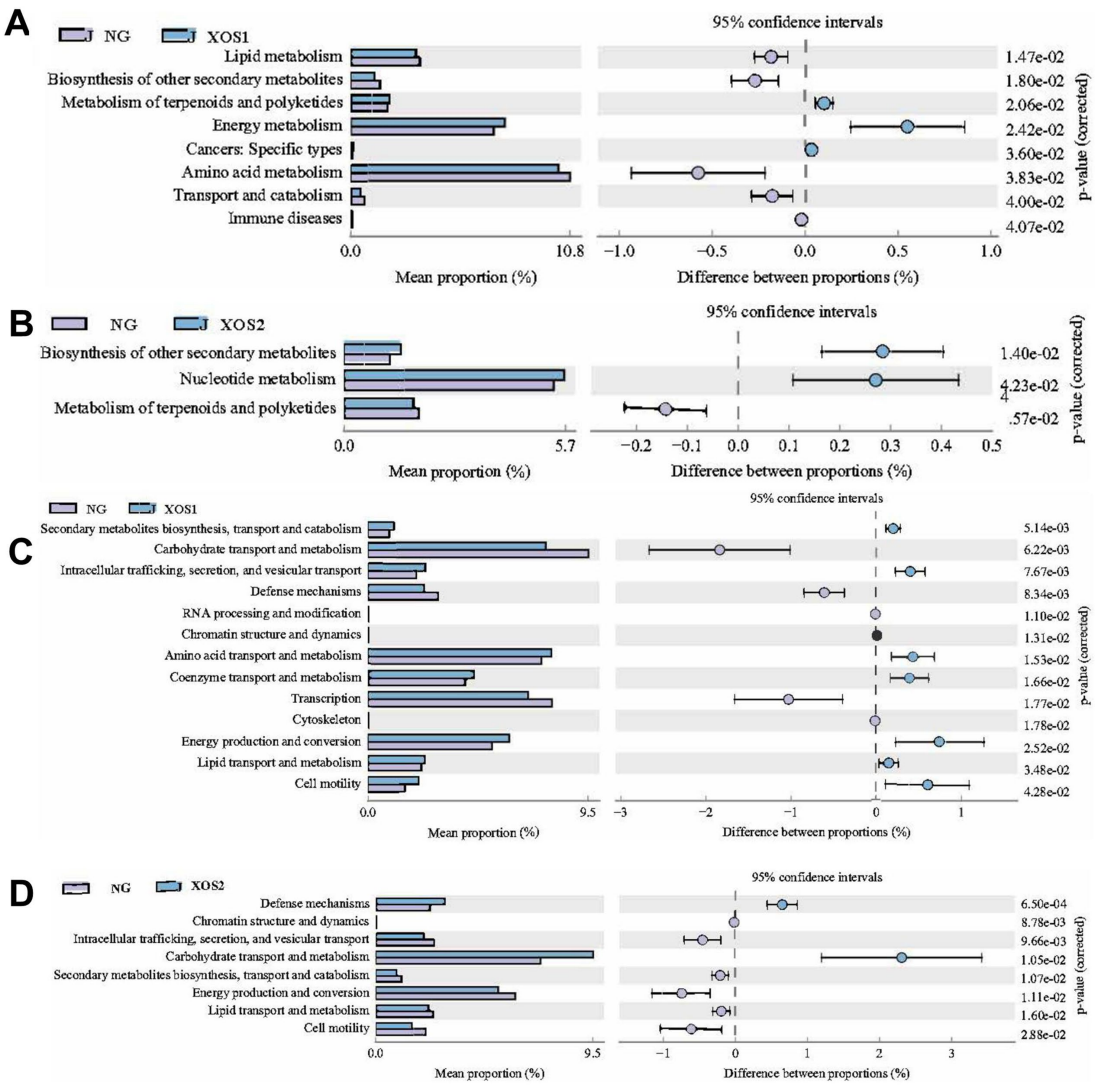
Based on KEGG analysis, the functional genes of the microbial community differed in the CK and XOS1 **(A)** and CK and XOS2 **(B)**.Based on COG analysis, the functional genes of the microbial community differed in the CK and XOS1 **(C)** and CK and XOS2 **(D)**.

Furthermore, PICRUSt software was used to predict the functional gene composition of samples by comparing the species composition information obtained from 16S sequencing data based on KEGG and COG database, so as to analyze the functional differences between different groups. Based on our analysis using the KEGG database, we identified that altered gut microbiotas were associated with various metabolic pathways, including organismal Systems, metabolism, Human Diseases, Genetic Information Processing, and Cellular Processes. To further understand the potential implication of these alterations, we screened and identified the significantly altered pathways that were correlated to the altered microbiotas. Compared with NG, lipid metabolism and biosynthesis of other secondary metabolites were significantly downregulated, with up-regulated metabolism of terpenoids and polyketides and energy metabolism in XOS1 (Figure 5). Meanwhile, the biosynthesis of other secondary metabolites and nucleotide metabolism were up-regulated in NG compared to XOS2, with a down-regulated metabolism of terpenoids and polyketides.

Furthermore, based on COG database, we found Secondary metabolites biosynthesis, transport and catabolism, Carbohydrate transport and metabolism, Intracellular trafficking, secretion, and vesicular transport. Defense mechanisms were evident differences between NG and XOS1, with significant differences in Defense mechanisms, Chromatin structure and dynamics, Carbohydrate transport and metabolism, and Energy production and conversion between NG and XOS2. These findings suggest that the gut microbiota May play a key role in regulating various metabolic processes.

## Discussion

The impact of xylooligosaccharides (XOS) on the gut microbiota of spleen deficiency constipation mouse models has been a subject of interest due to the potential therapeutic effects of prebiotics like XOS (Jain et al., 2015). In Traditional Chinese Medicine (TCM), spleen deficiency is associated with a variety of symptoms, including constipation, and is linked to an imbalance in the gut microbiota (Xiao et al., 2024). The modulation of the gut microbiota by XOS May contribute to the alleviation of constipation symptoms in spleen deficiency models. The novelty of using XOS in spleen deficiency constipation models lies in its potential to address the underlying cause of the constipation, rather than just treating the symptoms. By improving the gut microbiota balance, XOS May enhance the host’s digestive health and immune function, which are often compromised in spleen deficiency conditions.

In this study, fecal microbial composition and the diversity at different taxonomic units of the normal group (NG), the model group with spleen-deficiency constipation (SDC) and XOS treated groups that include XOS1 groups treated XOS 0.05 g/mL•d and XOS2 group treated XOS 0.1 g/mL•d were investigated using the 16S rRNA sequencing to understand the influence of XOS on intestinal microbiota of mice with spleen-deficiency constipation. The present study demonstrated that intestinal microbiota was changed after spleen-deficiency constipation and partially restored after the treatment with XOS. In the present study, the OTUs were applied to measure the abundance and diversity of bacterial population. Our results showed that the health and XOS-treated group had higher OTUs, and the XOS treated group had lower Chao index and Shannon index. These results indicated that the bacterial abundance and diversity decreased upon spleen-deficiency constipation but were restored by XOS treatment. The present research also found that the treatment May improve bacterial growth and proliferation. The results indicated that both the health and the XOS treated groups had abundant microbial species in their fecal samples. Firmicutes, Proteobacteria, and Bacteroidetes were predominant phyla in the four groups, and the abundance of *Firmicutes* in XOS1 group was the lowest, indicating that a high concentration XOS May increase the abundance of Firmicutes. However, the abundance of bacteria would decrease with the increase in XOS concentration. It was reported that Firmicutes were associated with fatty acid absorption and the abundance of Firmicutes was observed to be proportional to the obesity levels in the mice (You et al., 2020) and healthy horses were predominated by Firmicutes (68%) (Li et al., 2022).

In contrast to Bacteroidetes, Firmicutes metabolized sugar more efficiently, and it was favorable to energy resorption (Chen et al., 2021). These taxa are shown to be associated with obesity and intestinal diseases (Capetti et al., 2021). In porcine intestinal tract, XOS, which supported an enhancement of B*ifidobacteria* and *Lactobacilli* replication in the first stage of the fermentations, can be regarded as promising functional candidates suitable to act as distally fermentable substrates (Malykhina et al., 2017; Wegh et al., 2022). XOS can also be concluded to be an effective feed additive because it improves the quality of the eggshell by increasing the apparent digestibility of calcium and because it decreases the plasma GPT, cholesterol, HDL, and VLDL of laying hens (Bäckhed et al., 2004; Gomes et al., 2018). In broiler chickens, beneficial effects of XOS on broiler performance, when added to the feed, potentially can be explained by stimulation of butyrate-producing bacteria through cross-feeding of lactate and subsequent effects of butyrate on gastrointestinal function (Xu et al., 2019). In animals, many Bacteroidetes are beneficial to human beings and play important roles in maintaining intestinal functions, such as *Porphyromonadaceae* and *S24-7* (Mahaffee and Kloepper, 1997). Our results showed that the relative abundance of *Bacteroidetes* and *Porphyromonadaceae* were partially restored by the treatment with XOS of spleen-deficiency constipation, indicating XOS has the potential to recover intestinal bacteria and contribute to the treatment of spleen-deficiency constipation. In particular, XOS treatment evidently induced an increase of deferribacteres at the levels of *C_deferribacteres, F_deferribacteraceae, O_deferribacterales*, and *P_deferribacteres.* Deferribacteres are a group of bacteria that play a role in the human gut microbiome and have been associated with various health conditions, including gastrointestinal disorders (Stojanov et al., 2021). The presence of Deferribacteres in the gut is associated with a healthy and diverse microbial community. They can influence the host’s immune system and protect against certain pathogens by competing for resources and producing antimicrobial compounds (Jia et al., 2024). The use of XOS May indirectly affect the growth and metabolism of Deferribacteres by altering the nutritional content and pH value of the intestinal environment. This, in turn, could influence the occurrence and development of intestinal microbes, potentially contributing to the relief of symptoms related to spleen deficiency (Huang et al., 2024).

In summary, spleen-deficiency constipation impacted the abundance of intestinal bacterial populations, whereas XOS treatment readjusted them. The significance of this research is further highlighted by the potential for developing new therapeutic strategies for constipation that are rooted in the principles of TCM and supported by modern scientific evidence. This approach could offer an alternative or complementary treatment option for individuals suffering from constipation related to spleen deficiency, providing a more holistic and individualized approach to healthcare.

## Data Availability

The data presented in the study are deposited in the NCBI Sequence Read Archive (SRA project: PRJNA1166674).
